# Comparison of Microvessel Density and Growth Factor Levels Between Inner and Outer Prepuce in Distal Hypospadias*

**DOI:** 10.5152/tud.2025.24132

**Published:** 2025-05-21

**Authors:** Gaurav Prasad, Himanshu, Anjan Kumar Dhua, Seema Kaushal, Aswini Prabakaran, Shahnaz Ahmad Lone, Prabudh Goel, Sandeep Agarwala, Devendra Kumar Yadav, Hem Chandra Sati

**Affiliations:** 1 Department of Paediatric Surgery, All India Institute of Medical Sciences (AIIMS), New Delhi, India; 2 Department of Pathology, AIIMS, New Delhi, India; 3 Department of Paediatric Surgery, AIIMS, New Delhi, India; 4 Department of Biostatistics, AIIMS, New Delhi, India

**Keywords:** Growth factor levels, hypospadias, microvessel density

## Abstract

**Objective:**

Preputial flaps are frequently used in hypospadias repair, but the healing potential of preputial tissue remains a concern. This study aimed to evaluate the microvessel density (MVD) and growth factor receptor levels (VEGFR and TGF-βR) in the prepuce of patients with hypospadias compared to controls and to compare these parameters between the inner and outer preputial layers to determine their relative suitability for hypospadias repair.

**Methods:**

The study included children under 14 years of age undergoing primary distal hypospadias surgery as cases and individuals undergoing elective circumcision as controls. Specimens from the inner and outer prepuce were collected, and immunohistochemical staining was performed using CD31 antibodies to assess MVD and VEGFR and TGF-βR antibodies to evaluate growth factor levels. The staining intensity was semi-quantitatively analyzed using a histological scoring system.

**Results:**

Microvessel density was significantly higher in the inner prepuce than the outer prepuce in both cases and controls (*P* < .05), suggesting a potential advantage of inner prepuce tissue in hypospadias repair. Conversely, VEGFR levels were significantly higher in the outer prepuce in cases (*P* < .05), indicating different healing potentials between these layers. Transforming growth factor β receptor levels were significantly lower in both the inner and outer prepuce of controls compared to cases (*P* < .05). Other findings did not reach statistical significance.

**Conclusion:**

The higher MVD in the inner prepuce suggests it may be preferable for hypospadias repair. However, the differential VEGFR levels between the inner and outer prepuce highlight complex healing dynamics, emphasizing the need for further research to draw definitive conclusions.

**Main points**

- Inner prepuce shows higher microvessel density, indicating its potential advantage for use in neourethral reconstruction in hypospadias surgery.
- Growth factor receptor expression differs between inner and outer prepuce, with Vascular Endothelial Growth Factor Receptor higher in the outer prepuce, suggesting distinct tissue roles in healing and repair.
- Further research is needed to validate these findings, especially considering the variability in other growth factor levels and the need for larger sample sizes.

## Introduction

Hypospadias is a commonly occurring congenital anomaly^1^ that requires surgical correction to improve both the cosmetic appearance and functionality of the penis. Despite significant advancements in hypospadias repair techniques, finding a universal solution has proven challenging, and complications are common. Prepuce plays a crucial role in hypospadias repair, being used extensively as a flap in onlay and tubularized pedicled flaps, as well as in free grafts for tubularized incised plate repairs, intending to improve overall outcomes.^2^^-^ ^4^

The compromised healing potential of the prepuce and penile skin used for neourethral reconstruction can be evaluated through the assessment of microvessel density (MVD) using the panendothelial cell antigen CD31, as well as the examination of various growth factor levels. Microvessel density provides an objective measure of prepuce microvessels, including capillaries, venules, and arterioles, through immunohistochemical (IHC) markers like CD31.^5^ ^6^ Research has shown that children with hypospadias experience reduced MVD in the prepuce, along with changes in key growth factors such as vascular endothelial growth factor (VEGF) and its receptors (VEGFR), and epidermal growth factor receptor (EGFR).^7^ ^8^ Vascular endothelial growth factor, essential for angiogenesis and wound healing, is significantly diminished in hypospadiac prepuce compared to normal prepuce.^7^ ^9^ Moreover, decreased expression of the TGF-β receptor (TGFBR) in hypospadiac penile skin indicates potential healing difficulties due to its roles in immune modulation, endothelial adhesiveness, extracellular matrix synthesis, tissue repair, and collagen synthesis.^10^

Despite extensive documentation of the morphological and vascular anomalies in hypospadias, detailed insights into the molecular mechanisms of angiogenesis and wound healing in the prepuce remain limited. In particular, the roles of angiogenic factors such as VEGF—essential for reestablishing microcirculation under hypoxic conditions—and TGF-β, which modulates immune responses and extracellular matrix synthesis, warrant further exploration in the context of hypospadias repair.

Although previous studies have explored the expression of these growth factors, there is a lack of comprehensive evaluation regarding their influence on the different layers of the prepuce. This study aims to address this gap by systematically assessing the MVD in both the outer and inner layers of the prepuce while also estimating the expression of VEGFR and TGFBR in the epithelium of each layer. This investigation aims to determine if either the outer or inner layer exhibits superior healing potential compared to the other. To achieve this objective, a comprehensive comparison is made between the MVD and growth factor expression in the outer and inner layers of the prepuce in both normal and hypospadias subjects. By thoroughly evaluating these factors, this study seeks to provide valuable insights that can enhance surgical strategies and ultimately improve outcomes in hypospadias repair.

## Materials and Methods

Ethical approval was obtained from The All India Institute of Medical Sciences Ethics Board (IECPG-106/28.02.2019), and informed consent was obtained from the parents. Cases consisted of children under the age of 14 with distal hypospadias who were undergoing their first hypospadias surgery. Patients with a history of hormone therapy or treatment for local penile lesions were excluded from the study. Controls included patients undergoing elective circumcision without a history of phimosis or balanitis. All surgeries were performed by 2 surgeons (AD and PG).

### Sample Size Calculation

The sample size was calculated using Open Epi calculator (Dean AG, Sullivan KM, Soe MM. OpenEpi: Open Source Epidemiologic Statistics for Public Health, Version. www.OpenEpi.com, updated 2013/04/06), with a CI of 95%, power of 80%, and a sample size ratio of 1 (controls to cases).^11^ The sample size for cases was determined as 50 and controls as 50, resulting in a total sample size of 100. There were constraints imposed by the COVID-19 pandemic, which led to the suspension of routine outpatient department and operating theatre activities for a significant period during the study. Furthermore, restricted funds for procuring necessary reagents and strict time restrictions for completing the dissertation(this research was carried as part of a dissertation) hindered the ability to recruit and process the intended number of subjects; only 32 cases and 10 controls could be recruited.

### Surgical Method

During surgery, specimens measuring approximately 1.5 cm × 1.5 cm were obtained from the inner and outer layers of the prepuce at the 12 o’clock position.

### Histopathological and Immunohistochemical Evaluation

**Figure 1.**
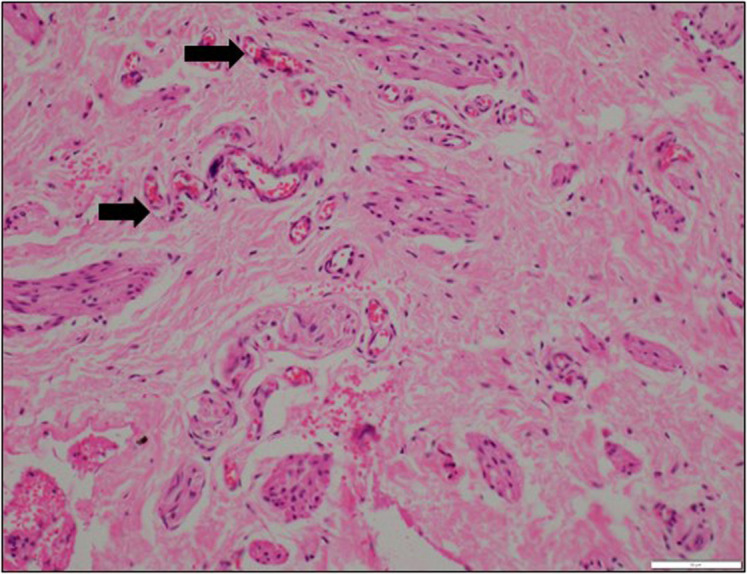
Hematoxylin and eosin (H & E) stained section of inner prepuce of a case with arrows highlighting the microvessels (×200).

Histopathology slides of inner and outer preputial sections stained with Hematoxylin and Eosin were reviewed (Figure 1). Additionally, thickness sections of 4-5 microns were cut on slides precoated with Poly L-Lysine for immunohistochemistry. The evaluation included assessing the MVD using CD31 (panendothelial cell antigen) (Invitrogen, PA5-29166, Rabbit Polyclonal antibody, Waltham, Massachusetts, United States) and the expression of VEGFR (Elabsciences, E-AB, 81493 Rabbit Monoclonal antibody, Houston, Texas, United States) and TGFβ Receptor (Santacruz, Biotechnology Inc., SC-74511, Mouse Monoclonal antibody, Dallas, Texas, United States).

The slides were deparaffinized and placed in a Tris(hydroxymethyl)aminomethane Buffered Saline (TBS) buffer of the desired pH. The tissue boundary was marked with a Dako pap pen. A primary antibody amplifier (biotinylated anti- mouse/anti-rabbit antibody) was added to the slides for 15 minutes. Horseradish peroxidase Streptavidin biotin was applied to each slide, then counter-staining was done with Harris Hematoxylin.

**Figure 2.**
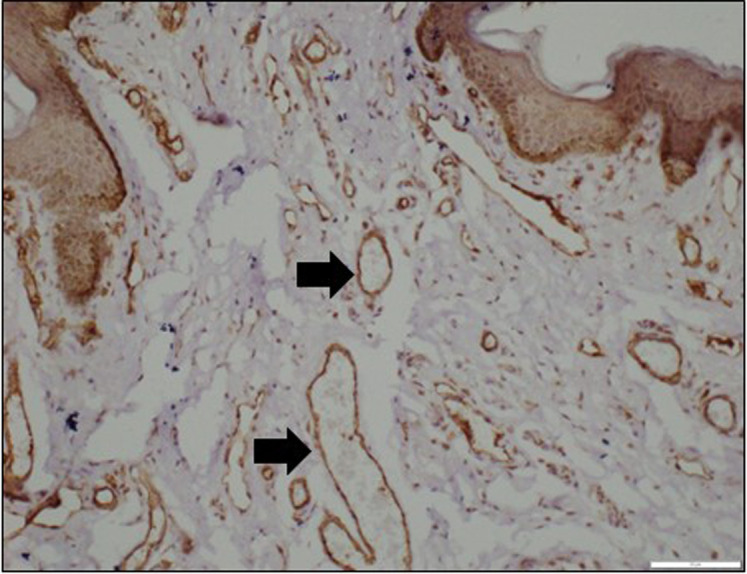
CD31 immunostaining of microvessels in a section of inner prepuce of a case with arrows highlighting the microvessels (×200).

Capillaries, small venules, and arterioles were examined to assess MVD (Figure 2). A light microscope (Olympus BX43, Tokyo, Japan) was used to identify regions with intense staining at low-power (×100) magnification. Two pathologists (SK and AP), blinded to the patient groups, performed this task. Subsequently, all microvessels stained positively with CD31 were counted across 5 randomly selected high-power (×200) fields within these identified regions of interest. The mean results were recorded for subsequent analysis.

**Figure 3.**
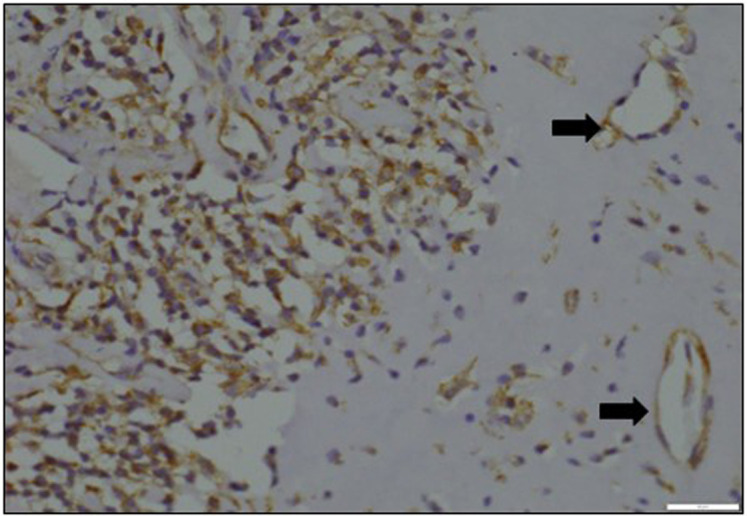
Vascular endothelial growth factor immunostaining of endothelial cells (black arrows) in a section of outer prepuce of a case (×400).

**Figure 4.**
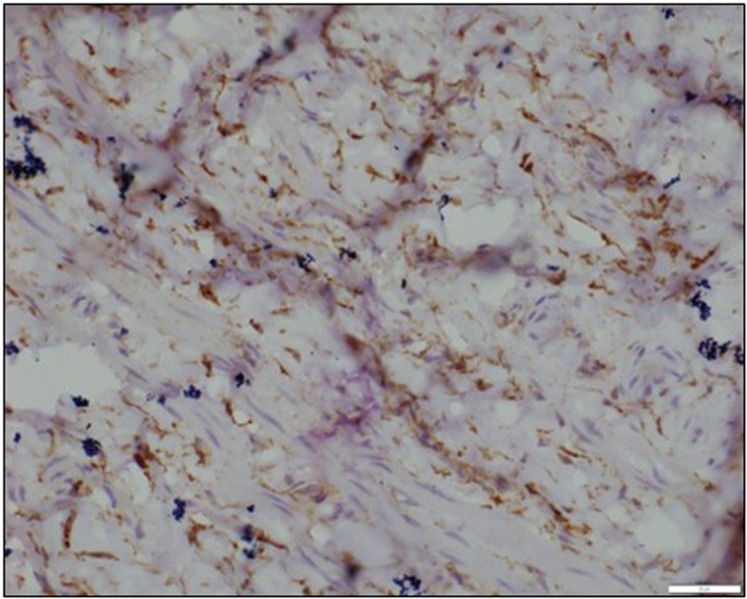
Transforming growth factor β immunostaining of endothelial cells (brown coloured) in a section of outer prepuce of a case (×400).

To evaluate the levels of VEGFR and TGFβ receptor staining intensity, a semi-quantitative approach employing an immunohistochemical histological scoring system (HSCORE) was utilized (Figure 3 and 4). The HSCORE ranged from 0 to 3+, with 0 indicating a negative result, 1+ representing weak staining intensity, 2+ denoting moderate intensity, and 3+ signifying strong staining intensity. The HSCORE calculation was determined by the formula [intensity of staining − x % of positivity]: (% of 0) × 0 + (% of 1+) × 1 + (% of 2+) × 2 + (% of 3+) × 3. The collective HSCORES ranged from 0 to 300.

Data analysis was conducted using STATA (StataCorp. 2015. Stata: Release 14. Statistical Software. College Station, TX: StataCorp LP, California, United States). The two-sample Wilcoxon rank-sum (Mann–Whitney) test was used to compare the results. A *P*-value of <.05 was considered statistically significant.

## Results

The study recruited a total of 32 cases and 10 controls.

### Microvessel Density

The MVD was higher in the inner prepuce (45.15 ± 10.74) compared to the outer prepuce (40.65 ± 7.62) in cases, and this difference was statistically significant (*P* = .0386). Similarly, the MVD was higher in the inner prepuce (47.2 ± 5.8) compared to the outer prepuce (36.7 ± 6.23) in controls, and this difference was also statistically significant (*P* = .0051). However, when comparing the MVD of the inner prepuce between cases (45.15 ± 10.74) and controls (47.2 ± 5.8), it was lower in cases, while the MVD of the outer prepuce was higher in cases (40.65 ± 7.62) compared to controls (36.7 ± 6.23). However, these differences were not statistically significant (*P* = .2312 and *P* = .1690, respectively) (Table 1).

### Vascular Endothelial Growth Factor Receptor

The median H-score of VEGFR was higher in the outer prepuce (181) compared to the inner prepuce (173.5) in cases, and this difference was statistically significant (*P* = .04). Similarly, it was higher in the outer prepuce (182.5) compared to the inner prepuce (180.5) in controls, although this difference was not statistically significant (*P* = .72).

When comparing cases and controls, the median H-score of VEGFR were lower in both the outer prepuce (173.5 for cases and 180.5 for controls) and the inner prepuce (181 for cases and 182.5 for controls) in cases of hypospadias compared to controls, but these differences were not statistically significant (Table 2).

### TGFβ Receptor

The median H-score of TGF-beta receptor was higher in the outer prepuce (73) compared to the inner prepuce (54.5) in cases. This difference was not statistically significant (*P* = .43). However, it was higher in the inner prepuce (34) compared to the outer prepuce (31) in controls, and this difference was also not statistically significant (*P* = .72) (Table 3). When comparing cases and controls, the median values of TGF-beta receptor were lower in both the outer prepuce (54.5 for cases and 34 for controls) and the inner prepuce (73 for cases and 31 for controls) in controls compared to cases, and these differences were statistically significant (*P* = .0081 and *P *= .0111, respectively).

## Discussion

The anatomical characteristics of the hypospadiac penis closely resemble those of a normal penis, with discernible differences on the ventral aspect, presenting as an underdeveloped foreskin, deficient urethra, and abnormalities in the urethral spongiosum. These observations imply inherent biological and structural deficiencies in the penile tissues affected by hypospadias.^12^ Extensive research has consistently identified variations in sensory innervation, levels of growth factors, vascular anatomy, and expressions of hormone receptors in hypospadiac penile tissues when compared to normal penile tissues, shedding light on the etiology of hypospadias and potential complications arising from surgical interventions.^13^

Arterial blood supply variations in hypospadiac prepuce have been examined by Perovic and Radojicic, highlighting a potential relationship between morphological abnormalities and vascular defects.^11^ Recent studies conducted by Celayir et al^14^ and Cağrı Savaş et al^15^ have focused on the expression of estrogen receptors and microvessel density (MVD), demonstrating a defective vascular pattern and decreased MVD in hypospadiac prepuce.^5^ These findings underscore the significance of comprehending the vascular anatomy in surgical outcomes and guiding decision-making concerning urethral reconstruction.

There is a scarcity of literature comparing MVD between the inner and outer preputial skin, and to the best of the authors’ knowledge, this is the first study to compare the levels of VEGFR and TGFβR between the inner and outer preputial skin.

In this study, the MVD of the inner prepuce was lower in cases compared to controls, although this difference was not statistically significant. However, the MVD of the outer prepuce in the controls was higher than in the cases and this was also not statistically significant. These findings do not align with the existing literature, where the MVD of the prepuce is lower in hypospadias cases than in controls, with statistical significance.^6^ ^15^ The MVD of the inner prepuce was higher than that of the outer prepuce for both cases and controls, and this finding was statistically significant. This finding aligns with the results of Elbakry et al,^16^ indicating that the healing potential of the inner prepuce is superior to that of the outer prepuce, and it should be preferentially be used for urethroplasty whenever feasible. The above finding reinforces the notion that the inner prepuce, with its denser microvasculature, may offer superior healing potential for urethroplasty. Clinically, this suggests that the inner prepuce could be preferentially selected for reconstructive procedures in hypospadias repair, potentially resulting in improved wound healing, lower rates of complications, and enhanced long-term surgical outcomes. By providing a more vascularized substrate, the inner prepuce may promote better tissue integration and resilience, which is crucial for successful neourethral reconstruction.

Elbakry et al^16^ stated that although MVD in the inner prepuce was slightly higher than in the outer prepuce, the wider lumen and well-developed wall of microvessels in the outer layer may compensate for this difference. However, they did not provide objective measurements of microvessel diameters or their wall thickness in their analysis. In contrast, microvessel density (MVD) offers a more standardized and quantifiable metric, allowing for a consistent and objective assessment of vascularization across different tissue samples. Thus, MVD remains a robust indicator of healing potential, particularly in the context of this study.

The study by el-Galley et al^8^ emphasized the significance of investigating growth factors such as VEGF, VEGFR, and TGF-βR to gain insight into the healing characteristics of the hypospadiac prepuce. In the study by Elbakry et al^16^, expression of EGFR was decreased in both layers, suggesting that either layer can be utilized for hypospadias repair without a significant preference.

The findings of this study related to growth factor levels provide significant insights into the healing potential and vascular characteristics of the hypospadiac prepuce. The statistically significant higher expression of VEGFR in the outer prepuce compared to the inner prepuce in cases suggests that the outer prepuce may have a more robust capacity for angiogenesis, which is essential for wound healing and tissue regeneration. This difference in VEGFR expression may indicate a differential role of the outer and inner prepuce in hypospadias repair, with the outer prepuce potentially having a more active role in processes driven by VEGF signalling.

The analysis revealed that TGF-βR expression is significantly higher in hypospadias cases compared to controls. TGF-β, known for its critical roles in immune modulation, endothelial adhesiveness, extracellular matrix synthesis, and tissue repair, typically increases with the onset of injury and ischemia. The elevated TGF-βR levels in hypospadiac tissue may therefore reflect a compensatory response to acute injury, distinguishing the active repair processes in these tissues from the more stable, quiescent state seen in normal prepuce.^17^ ^18^ Given that TGF-βR expression is reduced in chronic wounds, these findings suggest a pathological alteration in the wound healing environment that could contribute to the higher complication rates observed in hypospadias repair. This differential expression underscores the potential of TGF-βR as a biomarker for evaluating tissue repair dynamics in hypospadias, warranting further investigation.

This study’s findings align with existing literature that underscores the critical role of growth factor-mediated angiogenesis in tissue repair. VEGF, produced by endothelial cells and various other cell types, is indispensable for promoting endothelial cell survival and restoring perfusion through angiogenesis, especially under hypoxic conditions typical of acute wounds. Its reduced expression in chronic wounds raises concerns about inadequate healing in hypospadias. Similarly, TGF-β, through its receptor, orchestrates immune modulation, endothelial adhesiveness, and extracellular matrix remodeling, with increased receptor expression often serving as a compensatory response to ischemic injury.^7^ ^17^^-^ ^20^ The distinct alterations in VEGFR and TGF-βR expression observed in the hypospadiac prepuce samples provide further evidence of a disrupted healing environment.

Beyond individual roles, the coordinated interplay between growth factors is essential for an effective wound healing response. Vascular endothelial growth factor promotes endothelial cell proliferation and migration, driving angiogenesis and improving tissue perfusion, while TGF-β is crucial for collagen synthesis, wound contraction, and matrix remodeling. The integration of these pathways through their respective receptors underpins a complex healing environment. In this study, the higher VEGFR expression observed in the outer prepuce suggests an enhanced angiogenic potential that could support robust tissue regeneration. Conversely, the altered TGF-βR levels in hypospadias cases may reflect a compensatory response to acute injury, influencing collagen deposition and extracellular matrix organization.^17^ ^19^^-^ ^21^ This differential expression indicates that while the inner prepuce may be superior in terms of vascular density, the outer prepuce might contribute uniquely through its growth factor-mediated responses. These findings highlight the need for further molecular studies to dissect the dynamic interactions between VEGF and TGF-β signaling pathways in hypospadias repair, ultimately paving the way for targeted therapeutic interventions to improve surgical outcomes.

This study reveals that the results comparing the outer and inner prepuce are distinct for MVD and VEGFR. While MVD is significantly higher in the inner prepuce, VEGFR expression is notably higher in the outer prepuce, suggesting different functional roles for these tissue layers in hypospadias repair. However, not all findings in this study reached statistical significance, particularly in other growth factor assessments, which limits the ability to draw firm conclusions from those results. These discordant findings highlight the complexity of the tissue-specific healing potential and underscore the need for a more comprehensive analysis, including larger sample size and molecular studies, to fully understand the underlying mechanisms. Such studies are essential before any definitive recommendations can be made regarding the optimal use of preputial tissues in hypospadias surgery.

It is important to acknowledge certain limitations of this study. The small sample size may have affected the statistical power to detect significant differences in MVD, VEGFR, and TGF-β Receptor levels between the inner and outer preputial skin of the cases and controls. This limited sample size was due to the constraints imposed by the COVID-19 pandemic, limited funds and strict time restrictions for completing the dissertation. Future studies with larger cohorts are essential to validate these findings and improve their generalizability, ultimately enhancing the understanding of the molecular mechanisms in hypospadias repair. Furthermore, this study exclusively focused on distal cases, as sampling preputial tissue in proximal cases could impede hypospadias repair and be ethically inappropriate. Additionally, assessing biomarkers in the preputial skin may enhance the yield of the results obtained through immunohistochemistry. Prospective studies should be conducted to compare the clinical outcomes of hypospadias repair using the inner versus outer preputial skin.

In conclusion, this study reveals significant differences in microvessel density and growth factor receptor expression between the outer and inner prepuce, with the inner prepuce showing potential advantages for neourethral reconstruction. However, certain non-significant findings highlight the need for further research with larger sample sizes and molecular studies to optimize surgical outcomes in hypospadias repair.

## Supplementary Materials

Supplementary Material

## Figures and Tables

**Table 1.** Comparison of Microvessel Density

Variable	Case	Control	*P*
MVD Inner Prepuce	45.15 ± 10.74	47.2 ± 5.8	.2312
MVD Outer Prepuce	40.65 ± 7.62	36.7 ± 6.23	.1690
*P*	.0386	.0051	

Table 1. Table depicting the MVD (mean ± SD) of the outer prepuce and inner prepuce in cases and controls and the respective *P*-values.

MVD, microvessel density.

**Table 2.** Comparison of Vascular Endothelial Growth Factor Receptor Levels

Variable	Case	Control	*P*
VEGFR Inner Prepuce	173.5(44-270)	180.5(32-261)	.83
VEGFR Outer prepuce	181(29-268)	182.5(20-271)	.80
*P*	.04	.72	

Table 2. Table depicting the median H-score of VEGFR of the outer prepuce and inner prepuce in cases and controls and the respective *P*-values.

VEGFR, vascular endothelial growth factor.

**Table 3.** Comparison of Transforming Growth Factor β Levels

Variable	Case	Control	*P*
TGF-BetaR Inner Prepuce	54.5(20-196)	34(6-81)	.0081
TGF-BetaR Outer prepuce	73(15-179)	31(18-73)	.0111
*P*	.4375	.3071	

Table 3. Table depicting the median H-score of TGF β Receptor of the outer prepuce and inner prepuce in cases and controls and the respective *P*-values.

TGF β, transforming growth factor β.

**Supplementary Material 1.** Data used for analysis, including MVD values and H-score values for growth factor receptors (.xls file).

Serial Number	Case(1)/Control(0)	MVD-A	MVD-B	VEGFR-A	VEGFR-B	TGFβR-A	TGFβR-B
1	1	60	46	148	166	140	53
2	1	43	42	68	29	50	95
3	1	37	40	44	30	20	15
4	1	60	45	149	164	196	179
5	1	32	30	107	125	38	17
6	1	38	45	270	259	29	57
7	1	33	27	236	226	149	63
8	1	42	32	257	266	44	44
9	1	39	47	267	268	47	90
10	1	40	33	158	176	88	88
11	1	38	29	204	212	82	87
12	1	42	53	199	208	51	70
13	1	51	41	227	252	91	99
14	1	61	43	178	230	50	102
15	1	54	44	176	197	128	126
16	1	34	30	87	92	26	28
17	1	31	29	144	148	58	62
18	1	37	32	178	182	58	60
19	1	48	40	138	100	38	36
20	1	63	33	171	180	38	102
21	1	48	54	197	178	122	114
22	1	53	45	200	204	104	44
23	1	34	40	132	132	118	127
24	1	45	42	151	202	20	152
25	1	41	52	148	168	38	47
26	1	43	38	165	190	42	76
27	1	36	48	204	209	32	20
28	1	48	42	146	140	162	50
29	1	41	49	201	258	113	29
30	1	46	39	184	144	30	84
31	1	48	40	202	210	67	77
32	1	79	51	131	151	71	102
33	0	39	36	149	177	38	66
34	0	38	31	131	271	42	59
35	0	53	48	72	164	81	32
36	0	51	41	174	181	26	73
37	0	52	44	32	152	30	30
38	0	44	40	211	20	12	18
39	0	55	30	187	188	48	24
40	0	45	31	204	194	46	58
41	0	45	34	261	185	6	18
42	0	50	32	260	184	10	19

A = Inner Prepuce, B = Outer prepuce; MVD, microvessel density; VEGFR, vascular endothelial growth factor receptor; TGFβR, transforming growth factor β receptor.

## Data Availability

The data that support the findings of this study are available on request from the corresponding author.

## References

[1] Leung AKC Robson WLM. Hypospadias: an update. Asian J Androl. 2007;9(1):16 22. (doi: 10.1111/j.1745-7262.2007.00243.x) 17187155

[2] Prat D Natasha A Polak A Surgical outcome of different types of primary hypospadias repair during three decades in a single center. Urology. 2012;79(6):1350 1353. (doi: 10.1016/j.urology.2011.11.085) 22503767

[3] Mesrobian HGO Canning DA. Surgical technique for antegrade dissection of the preputial vascular pedicle during hypospadias repair. J Pediatr Urol. 2012;8(3):282 284. (doi: 10.1016/j.jpurol.2011.03.020) 21596623

[4] Mouravas V Filippopoulos A Sfoungaris D. Urethral plate grafting improves the results of tubularized incised plate urethroplasty in primary hypospadias. J Pediatr Urol. 2014;10(3):463 468. (doi: 10.1016/j.jpurol.2013.11.012) 24360521

[5] Yucel S Guntekin E Kukul E Comparison of hypospadiac and normal preputial vascular anatomy. J Urol. 2004;172(5 Pt 1):1973 6; discussion 1976. (doi: 10.1097/01.ju.0000142131.37693.05) 15540769

[6] Ceyhan L Cagri Savas MC Baspinar S Duman L Büyükyavuz BI. The correlation between preputial blood flow and microvessel density in distal hypospadias: A prospective clinical study. J Pediatr Urol. 2014;10(1):103 106. (doi: 10.1016/j.jpurol.2013.07.003) 23906986

[7] SOYER T AYVA EŞ ATASOY P ASLAN MK ÇAKMAK AM. Comparison of growth factor levels in patients with normal and hypospadiac prepuce. Turk J Med Sci. 2011;41(1):81 85. (doi: 10.3906/sag-1003-718)

[8] el-Galley RE Smith E Cohen C Petros JA Woodard J Galloway NT. Epidermal growth factor (EGF) and EGF receptor in hypospadias. Br J Urol. 1997;79(1):116 119. (doi: 10.1046/j.1464-410x.1997.22624.x) 9043509

[9] Fehrenbach A Pufe T Wittwer T Reduced vascular endothelial growth factor correlates with alveolar epithelial damage after experimental ischemia and reperfusion. J Heart Lung Transplant. 2003;22(9):967 978. (doi: 10.1016/s1053-2498(02)01157-9) 12957606

[10] Falanga V Qian SW Danielpour D Katz MH Roberts AB Sporn MB. Hypoxia upregulates the synthesis of TGF-beta 1 by human dermal fibroblasts. J Invest Dermatol. 1991;97(4):634 637. (doi: 10.1111/1523-1747.ep12483126) 1940433

[11] Perovic SV Radojicic ZI. Vascularization of the hypospadiac prepuce and its impact on hypospadias repair. J Urol. 2003;169(3):1098 1101. (doi: 10.1097/01.ju.0000052820.35946.99) 12576861

[12] Baskin LS Ebbers MB. Hypospadias: anatomy, etiology, and technique. J Pediatr Surg. 2006;41(3):463 472. (doi: 10.1016/j.jpedsurg.2005.11.059) 16516617

[13] Nazir Z Masood R Rehman R. Sensory innervation of normal and hypospadiac prepuce: possible implications in hypospadiology. Pediatr Surg Int. 2004;20(8):623 627. (doi: 10.1007/s00383-004-1244-1) 15449086

[14] Celayir S Eliçevik M Tireli G Dervisoğlu S Sander S. Expression of estrogen and androgen receptors in children with hypospadias: preliminary report. Arch Androl. 2007;53(2):83 85. (doi: 10.1080/01485010601166862) 17453687

[15] Cağrı Savaş M Kapucuoğlu N Gürsoy K Başpınar S. The microvessel density of the hypospadiac prepuce in children. J Pediatr Urol. 2011;7(2):162 165. (doi: 10.1016/j.jpurol.2010.04.017) 20627813

[16] Elbakry A Matar A Zalata K Zakaria A Al Atrash G. Microvasculature and healing potential of the inner versus outer preputial skin: preliminary immunohistochemical observations. Int Urol Nephrol. 2015;47(2):217 222. (doi: 10.1007/s11255-014-0881-0) 25409933

[17] Barrientos S Stojadinovic O Golinko MS Brem H Tomic-Canic M. Growth factors and cytokines in wound healing. Wound Repair Regen. 2008;16(5):585 601. (doi: 10.1111/j.1524-475X.2008.00410.x) 19128254

[18] Kaya M Soyer T Ayva S Cakmak M. Effect of penile tourniquet on growth factors in rat penile tissue. Eur J Pediatr Surg. 2009;19(4):236 240. (doi: 10.1055/s-0029-1215600) 19387925

[19] Leung DW Cachianes G Kuang WJ Goeddel DV Ferrara N. Vascular endothelial growth factor is a secreted angiogenic mitogen. Science. 1989;246(4935):1306 1309. (doi: 10.1126/science.2479986) 2479986

[20] O’Kane S Ferguson MW. Transforming growth factor Beta s and wound healing. Int J Biochem Cell Biol. 1997;29(1):63 78. (doi: 10.1016/s1357-2725(96)00120-3) 9076942

[21] Wu L Xia YP Roth SI Gruskin E Mustoe TA. Transforming growth factor-beta1 fails to stimulate wound healing and impairs its signal transduction in an aged ischemic ulcer model: importance of oxygen and age. Am J Pathol. 1999;154(1):301 309. (doi: 10.1016/s0002-9440(10)65276-5) 9916944 PMC1853440

