# Supplementary material for: Comparison of Microvessel Density and Growth Factor Levels Between Inner and Outer Prepuce in Distal Hypospadias*
Source: Urol Res Pract. 2025 May 21;51(1):1–6. doi: 10.5152/tud.2025.24132 (PMC12128352; doi:10.5152/tud.2025.24132)
Supplement: Supplementary Material [file supplementary_material.pdf]

Supplementary Material 1. Data used for analysis, including MVD values and H-score values for growth factor receptors (.xls file).

Serial Number	Case(1)/ Control(0)	MVD-A	MVD-B	VEGFR-A	VEGFR-B	TGFβR-A	TGFβR-B
1	1	60	46	148	166	140	53
2	1	43	42	68	29	50	95
3	1	37	40	44	30	20	15
4	1	60	45	149	164	196	179
5	1	32	30	107	125	38	17
6	1	38	45	270	259	29	57
7	1	33	27	236	226	149	63
8	1	42	32	257	266	44	44
9	1	39	47	267	268	47	90
10	1	40	33	158	176	88	88
11	1	38	29	204	212	82	87
12	1	42	53	199	208	51	70
13	1	51	41	227	252	91	99
14	1	61	43	178	230	50	102
15	1	54	44	176	197	128	126
16	1	34	30	87	92	26	28
17	1	31	29	144	148	58	62
18	1	37	32	178	182	58	60
19	1	48	40	138	100	38	36
20	1	63	33	171	180	38	102
21	1	48	54	197	178	122	114
22	1	53	45	200	204	104	44
23	1	34	40	132	132	118	127
24	1	45	42	151	202	20	152
25	1	41	52	148	168	38	47
26	1	43	38	165	190	42	76
27	1	36	48	204	209	32	20
28	1	48	42	146	140	162	50
29	1	41	49	201	258	113	29
30	1	46	39	184	144	30	84
31	1	48	40	202	210	67	77
32	1	79	51	131	151	71	102
33	0	39	36	149	177	38	66
34	0	38	31	131	271	42	59
35	0	53	48	72	164	81	32
36	0	51	41	174	181	26	73
37	0	52	44	32	152	30	30
38	0	44	40	211	20	12	18
39	0	55	30	187	188	48	24
40	0	45	31	204	194	46	58
41	0	45	34	261	185	6	18
42	0	50	32	260	184	10	19

A = Inner Prepuce, B = Outer prepuce; MVD, microvessel density; VEGFR, vascular endothelial growth factor receptor; TGFβR, transforming growth factor β receptor.
